# Differences in Temporal Relapse Characteristics Between Affective and Non-affective Psychotic Disorders: Longitudinal Analysis

**DOI:** 10.3389/fpsyt.2021.558056

**Published:** 2021-02-22

**Authors:** Sarah A. Immanuel, Geoff Schrader, Niranjan Bidargaddi

**Affiliations:** ^1^College of Medicine and Public Health, Flinders University, Adelaide, SA, Australia; ^2^Flinders Digital Health Research Centre, Flinders University, Adelaide, SA, Australia; ^3^Barossa Gawler Adelaide Hills Fleurieu Local Health Network, Adelaide, SA, Australia

**Keywords:** trajectory, hospitalization, relapse, trend, psychosis, affective disorders

## Abstract

**Objective:** Multiple relapses over time are common in both affective and non-affective psychotic disorders. Characterizing the temporal nature of these relapses may be crucial to understanding the underlying neurobiology of relapse.

**Materials and Methods:** Anonymized records of patients with affective and non-affective psychotic disorders were collected from SA Mental Health Data Universe and retrospectively analyzed. To characterize the temporal characteristic of their relapses, a relapse trend score was computed using a symbolic series-based approach. A higher score suggests that relapse follows a trend and a lower score suggests relapses are random. Regression models were built to investigate if this score was significantly different between affective and non-affective psychotic disorders.

**Results:** Logistic regression models showed a significant group difference in relapse trend score between the patient groups. For example, in patients who were hospitalized six or more times, relapse score in affective disorders were 2.6 times higher than non-affective psychotic disorders [OR 2.6, 95% CI (1.8–3.7), *p* < 0.001].

**Discussion:** The results imply that the odds of a patient with affective disorder exhibiting a predictable trend in time to relapse were much higher than a patient with recurrent non-affective psychotic disorder. In other words, within recurrent non-affective psychosis group, time to relapse is random.

**Conclusion:** This study is an initial attempt to develop a longitudinal trajectory-based approach to investigate relapse trend differences in mental health patients. Further investigations using this approach may reflect differences in underlying biological processes between illnesses.

## Introduction

Temporal characteristics of symptom onset have played an important role in the classification of psychiatric disorders. For example, Kraepelin's original distinction between manic depression and dementia praecox was based on the idea of manic depression, now known as bipolar disorder, being a recurrent illness with periods of complete recovery alternating with episodes of illness, while dementia praecox, now known as schizophrenia, having a chronic deteriorating course (1). With the introduction of specific treatments for bipolar disorder and schizophrenia, the outcome for both these disorders has considerably improved, although there is still debate about the extent of recovery that happens, even with treatment, particularly for schizophrenia. For example, Harrow et al. found in a 15 year follow up of patients with schizophrenia treated with contemporary interventions that while over 40% cumulatively had a period of recovery, this was followed in 60% by a period of symptom recurrence (2). Lang et al. similarly found in an extensive review of long-term outcome studies that schizophrenia had a generally poorer outcome than other diagnostic groups (3).

It has been argued that the distinction between outlook in schizophrenia and bipolar disorder may have neurological underpinnings. It has been shown that children who go on to develop schizophrenia (4) have evidence of cognitive and neurodevelopmental impairment and this is not evident in children who go on to develop bipolar disorder. These children who develop schizophrenia are also more likely to have a history of obstetric complications that could affect neurological development (5). Recently, several studies have attempted a finer grained analysis of natural history in schizophrenia in an attempt to better understand different illness trajectories and thereby better predict individual outcomes. For example, Ayesa-Arriola et al. described four different patterns of recovery in first episode psychosis based on symptoms on initial presentation (6). Velthoorst et al. compared illness trajectories and found multiple trajectories based on symptom patterns within each disorder (7). Patients with schizophrenia had more impaired trajectories, and those with mood disorders had better functioning trajectories. Such studies generally compare trajectories of distinct groups based on symptom clustering and or level of function, rather than examining any periodicity within the illness trajectory (8, 9). Periodicity, however, is important for diagnosing some affective disorders. Seasonal affective disorder and rapid cycling bipolar disorder are examples where diagnosis is made on the basis of a temporal relapse pattern characterized by predictability. Furthermore, evidence indicates people with affective disorders are more likely to have genetic polymorphisms associated with seasonal circadian disturbances (10). Additionally, within affective disorders as the number of relapses increases, there appears to be a shortening of time intervals between subsequent relapses (11). To our knowledge there have been no studies where patterns in time between relapse have been examined in non-affective psychosis.

We hypothesize, that while both affective and non-affective disorders are characterized by relapses, the temporal nature of relapse itself differs between these disorders, consistent with the differences in the underpinning biological processes and etiology. Specifically, affective disorder relapses are more likely to exhibit an inherent predictable trend pattern. Hospitalization is widely recognized as a useful proxy for reporting relapse when reporting in a naturalistic setting (12). Hence, we conducted our study on a large anonymized administrative health data set, taking rehospitalisation or presentation to the emergency department as an objective measure of relapse (13, 14). To compute the temporal nature of relapse, we applied symbolic series approach (15) on the time period between consecutive hospitalizations (gaps) for each individual. The symbolic series approach is useful for identification of predictable trend patterns within the time series while reducing inherent noise (16, 17). A predictable trend pattern is observed, when gaps are progressively increasing (relapsing less often or less frequently) or decreasing (relapsing more often or frequently) or remaining the same (regular). The output of symbolic series trend is a score between 0 to 1 with higher values indicating increasing observations of predictable patterns in the time series. We investigated for differences if any, in relapse trend score for patients with recurrent non-affective psychosis compared with patients with recurrent affective disorder.

## Materials and Methods

### Dataset

In this retrospective study, anonymized longitudinal records of adult patients (18 to 65 years), with diagnosed psychiatric disorders who presented to emergency departments, or were admitted to public hospitals in South Australia between January 1, 2007, and March 10, 2017 were collected from the South Australian Mental Health Data Universe. Permission to migrate anonymized records to Flinders University for research was obtained with the approval of the South Australian Department of Health through a project agreement.

Each record of relapse contained an anonymized patient identifier, age, gender, primary ICD-10 diagnosis block documented during each hospitalization, date and time of relapse or admission and the date and time of discharge from the hospital. Other details regarding patients such as family history were not available from this data set. While most patients had a single hospitalization with no relapse within the analysis time frame, there were patients who had up to eleven relapses. While a patient may be diagnosed with an affective disorder on their first admission, the diagnosis may change to a non-affective psychotic disorder on subsequent admissions and the converse may also occur. For the purpose of this study, the primary mental health ICD-10 diagnostic block documented at each relapse to the hospital was extracted and if it had changed between relapses, the most frequently occurring diagnostic block was tagged as the primary diagnosis for each patient. Based on this diagnostic label, two subgroups of patients were extracted for analysis—patients with ICD 10 non-affective psychotic disorders, with F codes (F20–F29) and patients with ICD 10 affective disorders with F codes (F30–F39) (18). Patients with F code diagnoses F20–F29 characteristically have symptoms such as hallucinations and delusions and respond to antipsychotic medication (19), while patients with affective disorder diagnoses F 30–F39 have mood disturbances and respond to mood stabilizing medications (20). More details on the patient inclusion criteria are outlined in the Supplementary Material.

### Relapse Trend Score

**Figure 1:**
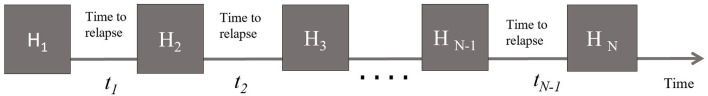
Schematic of a MH patient trajectory indicating hospitalizations and relapses.

**Figure 2:**
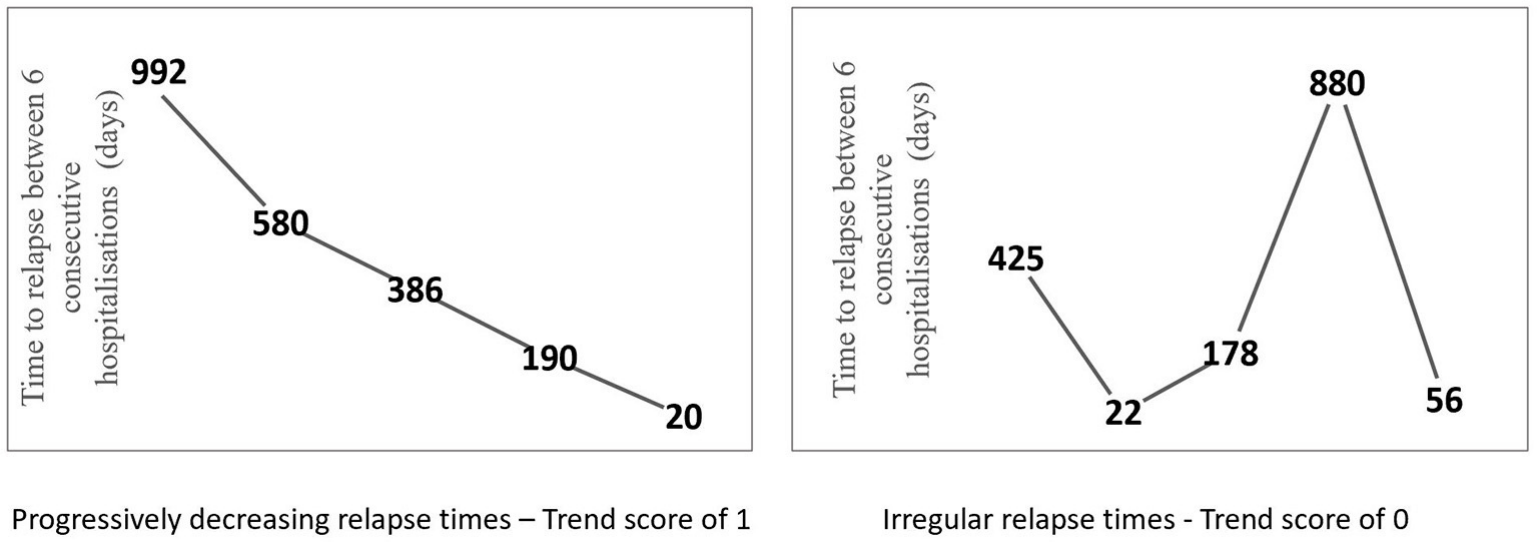
Relapse time trajectory of a patient with five relapses where the time to relapse is progressively decreasing between each hospitalization **(left)**. Relapse time trajectory of a patient with five relapses where the relapse times are irregular and do not follow a trend **(right)**.

The application of a symbolic series-based approach to compute the relapse trend score is explained in detail in the Supplementary Material. Briefly, this method captures the relapse onset temporal pattern for each individual based on the time gap between relapses and converts it into a score between 0 to 1 that describes the trend observed in these time gaps. An individual with a history of n relapses (*n* ≥ 4) has n-1 time units between the relapses (Figure 1). This series of time units, when iteratively grouped into three consecutive time units—with a shift in one time unit each iteration—will result in n-2 trend units. Each trend unit is a measure of direction of change between consecutive three time units and takes a score of 1 if consecutive time units form a predictable pattern, that is, are increasing or decreasing or remaining the same, and takes a score of 0 otherwise, in which case they are non-predictable or random patterns. The sum of these n-2 trend unit scores is then normalized within each cohort group based on the number of relapses. Thus, the temporal pattern score for an individual with n relapses is 1 when all n-2 trend scores are 1 and is 0 when none of the n-2 trend units scored is a 1, and anything in between will be a score between 0 to 1. A lower score suggests that relapse onset or time between hospitalizations is random, and a higher score suggests predictability over time. Figure 2 illustrates trend scores for two individuals with 6 relapses, one with a predictable trend and the other with unpredictable relapses. The above methodology is explained in more detail in the Supplementary Material.

### Statistical Analyses

Relapse trend score were computed using Matlab (The Mathworks Inc., Natick, MA) and data analyzed in SPSS statistical software v25. Patients were grouped into cohorts based on minimum number of relapses and regression models were developed within each cohort with age, gender and diagnosis as independent variables and the relapse trend score as the outcome variable. Linear and logistic regression were used, as appropriate, to explore the association between the outcome and the independent variables. Within the logistic model for association between relapse trend score and diagnostic information, the group that included patients with non-affective psychosis was used as the reference group and the affective disorder group was regressed against them. Odds ratios (ORs) and 95% CIs were obtained and used as a measure of effect size.

### Role of the Funding Source

The funders of the study had no role in study design, data collection, data analysis, data interpretation, or writing of the report.

## Results

The dataset included in the study had 8,658 subjects; 3,793 with non-affective psychosis and 4,865 with affective disorders. To analyze the relapse onset, a patient had to have at least four relapses so that the time gaps between them could be tested for a meaningful pattern. Hence, we retained only those patients from these two diagnostic groups, with more than four relapses and grouped them into cohorts based on their minimum number of relapses. The cohort size varied between 1,101 patients with four or more relapses and 43 patients who had 11 or more relapses. Table 1 shows demographics, distribution of diagnostic information and mean relapse trend scores within each cohort group.

**Table 1:** Distribution of age, gender, diagnostic information and relapse trend score among the patient cohorts.

**Number of relapses**	***N***	**Age**	**Female**	**Psychotic disorders**		**Affective disorders**	
		**Mean (SD)**	***N* (%)**	***N* (%)**	**Relapse trend score mean (SD)**	***N* (%)**	**Relapse trend score mean (SD)**
4	1,101	40.1 (14.9)	480 (43.6)	652 (59.2)	0.36 (0.2)	449 (40.8)	0.43 (0.4)
5	728	39.7 (15.0)	319 (43.8)	437 (60.0)	0.38 (0.1)	291 (40.0)	0.51 (0.4)
6	490	39.2 (14.8)	212 (43.3)	299 (61.0)	0.39 (0.2)	191 (39.0)	0.60 (0.4)
7	335	39.4 (15.2)	138 (41.2)	194 (57.9)	0.41 (0.3)	141 (42.1)	0.66 (0.4)
8	231	40.0 (14.5)	92 (39.8)	142 (61.5)	0.42 (0.3)	89 (38.5)	0.72 (0.3)
9	153	39.6 (14.7)	70 (45.8)	87 (56.9)	0.41 (0.3)	66 (43.1)	0.74 (0.3)
10	89	41.1 (15.1)	32 (36.0)	46 (51.7)	0.38 (0.2)	43 (48.3)	0.78 (0.3)
11	43	40.74 (14.9)	12 (27.9)	23 (53.5)	0.44 (0.3)	20 (46.5)	0.77 (0.2)

### Age and Gender

Univariate regression models showed that age was significantly associated with relapse trend score in almost all patient cohorts analyzed, while gender had no effect on the relapse trend score in most patient cohorts (Table 2). Overall, the direction of associations indicated that the odds of older subjects exhibiting a pattern or trend in their gaps between hospitalizations were higher than younger adults.

**Table 2:** Effect of age on the relapse trend score.

**Number of relapses**	**Coefficient (B)**	**SE**	***t***	***P*-value**	**95% CI of B**
11	0.3	0.35	0.8	0.412	[-0.4–1.01]
10	0.7	0.24	2.9	0.004	[0.23–1.17]
9	0.6	0.19	3.1	0.002	[0.22–0.96]
8	0.4	0.16	2.8	0.006	[0.13–0.78]
7	0.5	0.13	4.0	0.000	[0.26–0.77]
6	0.5	0.12	4.2	0.000	[0.25–0.71]
5	0.3	0.10	3.2	0.002	[0.12–0.52]
4	0.3	0.09	3.3	0.001	[0.12–0.51]

### Diagnosis

Within different cohort groups, diagnostic information was regressed against the relapse trend score. Logistic regression model fitting was statistically significant in all patient cohort groups (Table 3). Univariate analysis showed that patients with recurrent affective disorders were significantly more likely to exhibit a trend or pattern in time to relapse when compared to patients with non-affective psychosis (e.g., in cohorts with six or more relapses, OR 2.6, 95% CI 1.8–3.7, *p* < 0.001). This implies that the odds of a recurrent affective disorder patient exhibiting a trend or pattern in the time to relapse between his/her hospitalizations are 2.6 times the odds for a patient with recurrent psychosis. After adjusting for age, the recurrent affective disorder group still had a higher probability of having a trend or a pattern in time to relapse (Table 3).

**Table 3:** Association of patient diagnosis (covaried with age) with relapse trend score.

**Number of relapses**	**Coefficient (B)**	**SE**	**Wald χ^2^**	**Odds ratio**	**95 % CI of OR**
11	1.95	0.6	9.7	6.6	[2.0–21.5]^*^
10	2.13	0.4	22.5	9.9	[4.2–23.5]^**^
9	1.69	0.3	27.2	6.2	[3.3–11.7]^**^
8	1.55	0.3	33.5	5.0	[3.0–8.4]^**^
7	1.07	0.2	25.8	3.4	[2.2–5.0]^**^
6	0.87	0.2	24.9	2.6	[1.8–3.7]^**^
5	0.48	0.1	11.0	1.7	[1.3–2.2]^*^
4	0.19	0.1	2.3	1.3	[1.0–1.6]^*^

* p < 0.05;

** *p < 0.001*.

## Discussion

Our findings provide evidence that temporal patterns of relapse to hospitalization in non-affective psychotic disorders and affective disorders are significantly different. Longitudinal relapse trend analysis suggests that patients with affective disorders were more likely to have either increasing or decreasing times to relapse, while the time between relapse in non-affective psychotic disorders was more likely to be random. This distinction may reflect different underlying biological processes occurring in these conditions. For example, there may be more responsiveness to periodic environmental factors such as seasonal change in people with recurrent affective disorder (21). The biological processes behind non-affective psychosis may be less responsive to circadian rhythms and perhaps more likely to be affected by randomly occurring environmental, biological or social stress. Our findings are of note in view of concern that cross sectional categorical diagnostic systems such as DSM V define syndromes as heterogeneous in terms of etiology and treatment response (22). It is based on such concern the Research Domain operational Criteria (RoDC) (23) that considers classification of psychiatric disorders on the basis of biological mechanisms, endophenotypes and biomarkers. However, in our study, patients in the broad cross-sectional diagnostic categories of affective disorder or non-affective psychosis derived from administrative hospital data, had significantly different patterns of changes in rate of relapse over time.

This is the first study to use an entropy-based approach to elucidate how temporal patterns of relapse differ between different psychiatric diagnoses. Entropy based approaches have been applied to analyze patient trajectories in finding treatment benefits, exploring relapse in placebo-controlled trials, and monitoring physical activity trends after rehabilitation. Haimovich et al. conducted an analysis of condition-specific hospital utilization rates using a clustering based computational approach and demonstrated that a substantial proportion of medical conditions exhibit seasonal variation in hospital utilization (19). This is a simple, coarse grained technique, that is robust in capturing predictability in longitudinal time-series. There are a limited number of studies in the literature where mathematical approaches have been applied to psychiatric readmission data to look for associations with clinical, environmental and health system characteristics. In particular, temporal trends or patterns in repeated admissions based longitudinal mental health patient trajectories have not been investigated previously.

Our study had several limitations. We used the relatively broad F-code categories of the ICD-10 diagnostic system to categorize patients. Unfortunately, the coding of the derived dataset that we received from the South Australian Mental Health Data Universe database system did not allow for further distinctions to be drawn between individual affective disorder categories within F30–F39 such as bipolar disorder and individual psychotic disorder categories within F20–F29 such as schizophrenia. A further subgroup analysis with the raw data presented to demonstrate heterogeneity if any within the F20 and F30 cohorts will be a future scope of this study. For example, the demonstration of a distinct set of affective diagnoses associated with a decreasing interval of time between relapse would be of interest. Another limitation relates to our definition of relapse which included only hospital and emergency department contacts. The inclusion of outpatient records of recurrence may have captured a greater number of less extreme relapses and thus affected our findings. A further limitation may relate to how we categorized patients into the affective and psychotic groups, particularly those who had different diagnoses when admitted at different times. Our decision to use the most common diagnosis, however, would conceivably have made the groups more heterogeneous and thus would have reduced the likelihood of us finding any distinction between the two categories in terms of illness trajectories.

## Conclusion

To conclude, the presented approach is a proof of concept study toward longitudinal analysis of time trajectories in hospitalization data. We have woven together multiple hospitalizations of mental health patients and captured the trend in the time between each relapse. Temporal trends in relapse using time stamps along a mental health patient trajectory have not been investigated previously. This is a novel approach and a first step toward longitudinal trajectory-based approach to investigate relapse in mental health patients. Further investigation of patterns in mental health trajectories could provide insights into utilization of acute services over time and identify which individuals are at increased risk of readmissions.

## Data Availability Statement

The raw data supporting the conclusions of this article will be made available by the authors, without undue reservation.

## Ethics Statement

Ethical review and approval were not required for the study on human participants in accordance with the local legislation and institutional requirements. Written informed consent for participation was not required for this study in accordance with the national legislation and the institutional requirements.

## Author Contributions

SI study design, literature search, figures, data collection, data analysis, data interpretation, writing and revision of MS. GS literature search, data interpretation, writing and revision of MS. NB study design, data analysis, data interpretation, writing and revision of MS. All authors contributed to the article and approved the submitted version.

## Conflict of Interest

The authors declare that the research was conducted in the absence of any commercial or financial relationships that could be construed as a potential conflict of interest.
