# Supplementary material for: Differences in Temporal Relapse Characteristics Between Affective and Non-affective Psychotic Disorders: Longitudinal Analysis
Source: Front Psychiatry. 2021 Feb 22;12:558056. doi: 10.3389/fpsyt.2021.558056 (PMC7938319; doi:10.3389/fpsyt.2021.558056)
Supplement: Supplementary file 1 [file Data_Sheet_1.PDF]

Differences in temporal relapse characteristics between affective and non-affective psychotic disorders: Longitudinal analysis

Sarah A Immanuel[1,2], Geoff Schrader[1], Niranjan Bidargaddi[1,2]

[1]College of Medicine and Public Health, Flinders University, Adelaide, Australia

[2]Flinders Digital Health Research Centre, Flinders University, Adelaide, Australia

Correspondence to:

Dr Sarah Immanuel

College of Medicine and Public health

Flinders Digital health Research Centre

Flinders at Tonsley

1284 South Rd, Clovelly Park SA 5042

Sarah.immanuel@flinders.edu.au

Ph: +61 401 873 573

Supplement Material

Data

Two subgroups of patient data were extracted from the SA Mental Health Data Universe and used in the analysis presented. They were patients with non-affective psychotic disorders (F20-F29) and patients with affective disorders (F30-F39). Each record of relapse or admission into the hospital contained an anonymized patient identifier, age, gender, primary ICD-10 diagnosis block documented during hospitalization, date and time of relapse or admission and the date and time of discharge from the hospital.

Age and Diagnostic information

Age of patients was calculated using their data of birth and the start date of their first hospitalisation. When multiple hospitalisations are weaved together to form a patient trajectory, the trajectory contains a sequence of diagnostic information codes – one from each hospitalisation. These diagnostic codes could be the same on all hospitalisations but often changes between hospitalisations, especially when patients present with physical or mental health related comorbid conditions. If there were 'N' number of hospitalisations in a trajectory and if each hospitalisation had a different diagnostic code, then the number of unique codes would be N while if all hospitalisations had the same diagnostic code, then the number of unique codes would be one. In our analysis, if diagnostic information in each hospitalisation changed along a patient's trajectory, we used the most common or frequent diagnostic block or code along a trajectory as the patient's diagnostic information.

Patient trajectory

All hospitalisations for each patient were linked together in chronological order to form a patient trajectory. Each trajectory thus consists of one or more hospitalisations separated by the time to relapse or time gaps between the hospitalisations (Figure 1). These gaps represent the time spent by the patient in the community setting, without receiving hospital care.

Relapse time series

In patients who had more than one hospitalisation, the time gap between their hospitalisation was calculated in days using the end date of a given hospitalisation and the start date of the subsequent hospitalisation. For each subject, the time between the hospitalisations provide a relapse time series. To describe the dynamic aspect of this time series, a symbolic series approach was used.

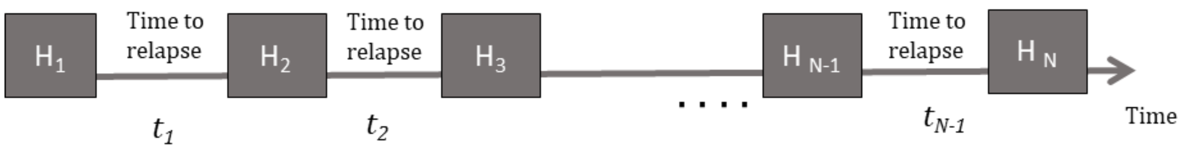

Figure A1. Schematic of a MH patient trajectory indicating hospitalizations and relapses

Patient trajectories are given by

$$P_j = \{ H_1, t_1, H_2, t_2, H_3, \dots\ t_{n-1}, H_n\}$$

H_i – Hospitalisations; t_i – Time to relapse between hospitalisations H_i and $H_{i+1;}$

The time to relapse between each hospitalisation episodes form a unique relapse time series for each patient, given by

$$T\ (P_j) = \{t_1, t_2, t_3, \dots, t_{n-1}\}$$

T – time series of time to relapse

j = 1 to J where J is the patient cohort size

Construction of symbolic sequences

The idea behind the symbolic dynamics approach is to symbolise this inter-hospitalisation relapse time series of each patient. Each *T(Pj)* is fitted into a time-grid which is divided into several equidistant steps. In our study, the levels or steps were fixed as 6 months. For a given $T\ (P_j)$, each t_i in the time series is fitted into a symbol based on the level or the band that they fit into.

For example, in a patient, if the time to relapse between two hospitalisations is between less than 6 months, that relapse is assigned a symbol '1' and if the relapse time is between 6 to 12 months, the relapse is assigned a symbol '2' and so on. Figure 2a shows an example of a patient trajectory with seven hospitalisations i.e. six gaps or relapses between hospitalisations. The gaps in months have been fitted into symbolisation levels based on steps of 6 months (left panel) and then converted into symbols (right panel) based on the symbolisation levels that the gap duration fits into. For instance, gap 1 was ~28 months and hence fell into level 5 and both gap 2 and gap 3 being 7 to 10 months; fell into level 2 and so on. Thus, the relapse time series of each patient trajectory was converted into a symbolic series as demonstrated in the Figure 2.

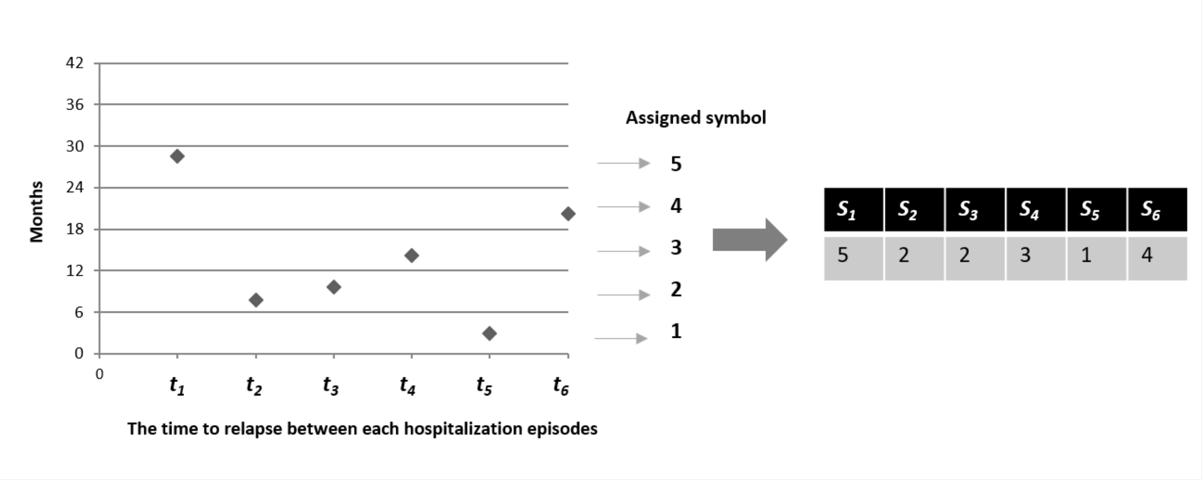

Figure A2: Example patient trajectory with seven hospitalisations. Left panel shows the symbolisation approach on the gap time series and the right panel shows resulting symbolic sequence ($S_1S_2S_3S_4S_5S_6 = 522314$).

Conversion of the symbolic sequences into three symbol words

The next step in the methodology was to convert the symbolic sequences into words. Three adjacent symbols in a symbolic series formed a word (length k =3) and then the process was repeated by shifting one symbol to the right. For example, combination of $S_1S_2S_3$ results in a three-symbol word W_1; Combination of $S_2S_3S_4$ makes a three-symbol word W_2 and so on. From the above example in Figure 2, W_1 is 522; W_2 is 223; W_3 is 231 and so on.

Coding words based on their pattern

Each three-symbol word is then coded into a number between 1 and 9. This is based on the pattern followed by the three symbols that constitute a word. A three-symbol word showing a decreasing ramp (monotonic decrease) or an increasing ramp (monotonic increase) or a constant line (regularity) as their pattern were considered as reflectors of a trend in the gap time series (coded as 1, 2 and 9 respectively) (Figure 3). Other combinations were considered indicators of fluctuations or variations without any trend (coded 3, 4, 5, 6, 7, 8 etc.). Each word was thus coded into an indicator that ranges between 1 and 9 based on the pattern followed by the three symbols that constitute the word. Using the three-symbol words from Figure 2 as examples, W_1 is 522, the relapse time decreased and then remained constant, hence forms a pattern similar to pattern 6 in Figure 3; W_2 is 223, relapse time remained constant and then increased, hence forms a pattern similar to pattern 5 in Figure 3; W3 is 231, relapse time increased and then decreased, hence forms a pattern similar to pattern 4 in Figure 3 and so on. After all possible word combinations are coded, the number of trend indicators of interest (1, 2 or 9) arising out of the coding process are counted and quantified as the relapse trend score. This is explained in the following section.

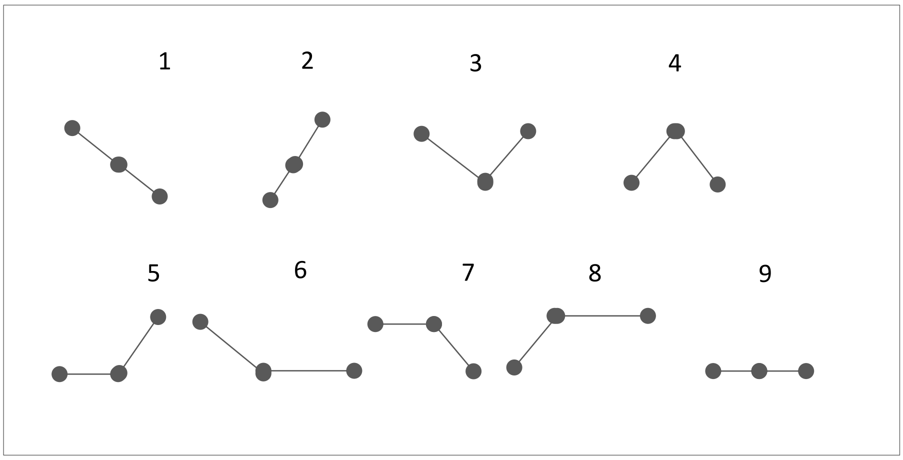

Figure A3: Possible patterns of three symbols in a word and their corresponding codes. Patterns described by codes 1, 2 and 9 are indicators of monotonic increase, monotonic decrease or constancy in the relapse times between patient hospitalisations and are considered indicators of a trend.

Relapse Trend Score

The occurrence of trend indicators of interest (1, 2 or 9) are quantified to compute the relapse trend score. To explain this process, consider the example in figure 4, that shows the symbolic sequence derived from eight relapses, in a patient with nine hospitalisations, using the approach explained in previous sections.

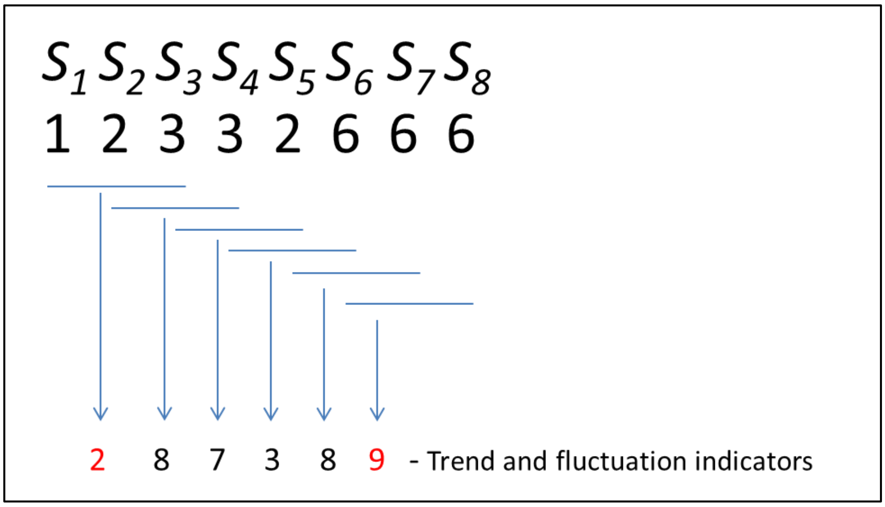

Figure A4: Transformation of an example symbolic time series into three symbol words and labelling each word with a code based on their pattern as defined in Figure 3.

Using this example of a patient trajectory with nine (n=9) hospitalisations, there would be eight (n-1) relapses and hence eight symbols in the symbolic sequence. Grouping these eight symbols into words of length k=3 using the above-mentioned approach would result in six (n-3) words W_1 to W_6. If all six (n-3) words are coded as either 1, 2 or 9 then there is maximum trend: score is 6 out of 6 i.e., 100% trend. Say if 5 words out of 6 were coded as 1, 2 or 9, then the score is 5 out of 6 i.e., 83% trend and so on. If none of the words received a score of 1, 2 or 9 then the trend in gap score is zero. In figure 4, out of the six three symbol word combinations, W_1 - $S_1S_2S_3$: 123 follows pattern coded as 2 in figure 3 (increasing trend) and W_6 - $S_6S_7S_8$: 666 follows pattern coded as 9 in figure 3 (constant trend). Only these two words indicate trend and other words capture fluctuations, thus the score is two out of six (33%).

The above methodology was applied on the relapse time between hospitalisations in each patient trajectory, with four or more relapses, and a relapse trend score is computed using their relapse time series. This score was the key outcome variable of interest in this study.
